# Is protein intake saturated at doses recommended by the feeding guidelines for critically ill patients?

**DOI:** 10.1186/s13054-018-2149-z

**Published:** 2018-09-22

**Authors:** Jan Gunst, Michael P. Casaer

**Affiliations:** Clinical Division and Laboratory of Intensive Care Medicine, Department of Cellular and Molecular Medicine, KU Leuven, Herestraat 49, 3000 Leuven, Belgium

The ideal protein intake for critically ill patients is still debated [1]. Guidelines recommend minimum doses of 1.2–1.3 g protein/kg/day for non-obese critically ill patients and doses up to 2.0–2.5 g/kg ideal body weight (IBW)/day for obese critically ill patients [2]. Yet, achieved intakes often do not meet the recommended targets and several randomized controlled trials have shown absence of clinical benefit and increased ureagenesis by strategies that increased amino acid administration to critically ill patients [1]. A recent mechanistic study implicated amino acid-induced glucagon secretion as a potential mediator of amino acid catabolism, which may drive a futile cycle [3].

Recently, van Zanten et al. [4] studied the feasibility of a high-protein diet in obese critically ill patients. Patients in the intervention group received, on average, 1.49 g protein/kg/day, compared to 0.76 g/kg/day in the control group. As in previous studies, patients in the high-protein group had significantly higher urea levels and increased urinary nitrogen excretion [1]. In critical illness, the amount of exogenous protein net degraded into urea may be substantial. A secondary analysis of the EPaNIC study estimated that over 2 weeks, almost two-third of the supplementary amino-acids provided by 5-6 days of early-PN were net wasted in urea, thereby potentially prolonging the need of renal-replacement-therapy [5].

From the data provided by van Zanten et al., it is not possible to exactly calculate the fraction of extra-delivered proteins that were net wasted in ureagenesis. However, a rough calculation (similar to in the online supplement of [5]) suggests that, over 5 days, also about two-thirds of the extra proteins could be wasted. Indeed, estimating a median difference in protein intake accumulating up to 3.7 g/kg IBW at day 5 (based on the figure) and an (ideal) body weight of 88 kg, approximately 325.6 g extra proteins were delivered to the high-protein group over 5 days, which corresponds to 52 g nitrogen. Assuming that the reported urinary nitrogen excretion corresponds to the average nitrogen excretion per day, and that the cumulative nitrogen excretion over the first 5 days corresponds to 5× that value, the supplementary urinary nitrogen loss in the high-protein group over 5 days is estimated to be 5 × 6 g or 30 g. Furthermore, assuming equal urea levels at baseline, taking into account a difference of 4.2 mmol/l blood urea nitrogen (BUN) reported at day 5 (=25.2 mg/dl urea) and a urea distribution volume of 0.6× body weight, the additional nitrogen converted to BUN and not yet excreted in the urine amounts to up to 6.2 g at day 5. Hence, supplementary nitrogen loss over 5 days would correspond to 69.6% of the supplementary proteins provided (36.2 g extra nitrogen loss of the 52 g supplementary nitrogen provided).

We suggest that the authors calculate the cumulative net amount of nitrogen retained (or wasted) at several time points based on the individual patients’ data. If such a calculation confirms our estimation, this would suggest that increasing protein delivery in the acute phase of critical illness up to the recommended doses for critically ill patients may be ineffective and potentially harmful. Furthermore, although hypothesis-generating, the authors may—based on their dataset—identify a time point at which catabolism becomes less resistant to feeding.

## Abbreviations

BUN: Blood urea nitrogen
IBW: Ideal body weight
